# Analysis of the Potential Genetic Links between Psoriasis and Cardiovascular Risk Factors

**DOI:** 10.3390/ijms22169063

**Published:** 2021-08-23

**Authors:** Dorota Purzycka-Bohdan, Anna Kisielnicka, Michał Bohdan, Aneta Szczerkowska-Dobosz, Marta Sobalska-Kwapis, Bogusław Nedoszytko, Roman J. Nowicki

**Affiliations:** 1 Department of Dermatology, Venereology and Allergology, Medical University of Gdansk, 80-214 Gdansk, Poland; a.kisielnicka@gumed.edu.pl (A.K.); adobosz@gumed.edu.pl (A.S.-D.); bned@gumed.edu.pl (B.N.); rnowicki@gumed.edu.pl (R.J.N.); 2 First Department of Cardiology, Medical University of Gdansk, 80-211 Gdansk, Poland; michal.bohdan@gumed.edu.pl; 3 Biobank Lab, Department of Molecular Biophysics, Faculty of Biology and Environmental Protection, University of Lodz, 90-231 Lodz, Poland; marta.sobalska@biol.uni.lodz.pl; 4 Invicta Fertility and Reproductive Centre, Molecular Laboratory, 81-740 Sopot, Poland

**Keywords:** psoriasis, comorbidities, cardiovascular risk, genetic background

## Abstract

Cardiovascular risk factors are one of the most common comorbidities in psoriasis. A higher prevalence of hypertension, insulin resistance and type 2 diabetes, dyslipidemia, obesity, metabolic syndrome, depression, as well as cardiovascular disease was confirmed in psoriatic patients in comparison to the general population. Data suggest that psoriasis and systemic inflammatory disorders may originate from the pleiotropic interactions with many genetic pathways. In this review, the authors present the current state of knowledge on the potential genetic links between psoriasis and cardiovascular risk factors. The understanding of the processes linking psoriasis with cardiovascular risk factors can lead to improvement of psoriasis management in the future.

## 1. Introduction

### 1.1. Psoriasis—Short Disease Outline

Psoriasis is a common, chronic inflammatory disease of the skin characterized by the formation of scaling plaques. The combination of immunological, genetic, and environmental factors plays an important role in its etiology [1,2]. Multiple genetic loci predispose to increased psoriasis susceptibility. The *PSORS-1* region is accountable for 30–50% of psoriasis heritability [1]. New genomic studies are strongly required to reveal possible hypothesis-free genetic determinants, single nucleotide polymorphisms (SNPs), and microarrays involved in psoriasis pathogenesis [3]. The role of the immune system, particularly Th1 and Th17 lymphocytes as well as tumor necrosis factor alpha (TNF-α), IL-17, IL-12, and IL-23, has been regarded as crucial in the development of psoriatic lesions [4]. Due to inflammatory background, psoriasis is recognized as a systemic disease. Similar risk factors as in psoriasis are considered to play an important role in the development of concomitant disorders.

### 1.2. Psoriasis and Comorbid Diseases—Clinical Data and Epidemiology

The exacerbation of psoriasis is associated with the activation of inflammatory processes not only within cutaneous lesions but also in the other tissues. The complex interactions between immune cells and cytokines in psoriasis may alter the occurrence of autoinflammatory diseases [5]. The common psoriasis comorbidities include cardiovascular risk factors (Table 1). Higher prevalence of hypertension (HT), insulin resistance and type 2 diabetes mellitus (DM-2), dyslipidemia, obesity, metabolic syndrome (MS), and depression was confirmed in psoriatic patients in comparison to the general population [6]. Obesity seems to be a crucial driving force for the development of psoriasis comorbidities [7]. The genetic background leads to greater susceptibility of psoriatic patients to metabolic disorders and cardiovascular disease (CVD). This hypothesis was verified in a large cross-sectional study on Danish twins, which indicated a common genetic etiology for psoriasis and obesity [8]. Furthermore, not only genetic predisposition but also various environmental and immunological factors may increase an individual’s cardiovascular risk. These factors range from infections, gut dysbiosis, and air pollution to behavioral aspects such as diet, physical activity, and smoking [9].

### 1.3. Psoriasis and Concomitant Cardiovascular Risk Factors—Clinical Consequences

Psoriasis comorbidities may affect the severity of the disease, lead to an early onset of symptoms, and decrease quality of life, which lead to reduced life expectancy. According to recent data, severe psoriasis is associated with an increased risk of death, which is mainly due to cardiovascular events [10]. Infections, kidney disease, and dementia have been also highlighted as additional risk factors increasing mortality among psoriasis patients. Obesity is considered as a significant negative prognostic factor in response to the therapy in psoriasis [11,12]. Early treatment of comorbidities, particularly obesity, may reduce the severity of psoriasis and therefore the risk of psoriatic therapy-related clinical consequences. There is evidence that psoriasis and systemic inflammatory disorders may originate from the pleiotropic mechanisms of interactions with many genetic pathways. Therefore, establishing a genetic linkage between psoriasis and cardiovascular risk factors may contribute to the novel approach to psoriasis treatment.

## 2. The Role of Inflammatory Molecules and Mechanisms in Psoriasis-Associated Diseases

### 2.1. Production of Inflammatory Factors is Mostly Regulated by Gene Transcription

Understanding the molecular processes may lead to the successful exploration of novel genetic loci and gene-to-gene interactions. Genetic studies have confirmed the interplay between the inflammatory pathways, gene polymorphisms, and the origin, course, and severity of the diseases. Furthermore, in the case of complex inflammatory disorders (such as CVD, HT, obesity, or psoriasis), it is important to consider the combination of various gene polymorphisms due to their cumulative and potentializing effect on the disease predisposition [13]. For example, an adipose tissue TNF-α messenger RNA (mRNA) correlates with BMI, percentage of body fat, and hyperinsulinemia [14]. TNF-α polymorphism at −308 gene locus nearby its promoter region facilitates the gene transcription, and thus, prominent TNF-α overproduction involved in inflammatory diseases origin [15]. Currently, long non-coding RNA (lncRNA) is considered as a gene transcription regulator of the inflammatory response pathways [16].

Caspase recruitment domain-containing protein 14 (CARD14) is an intracellular scaffold protein that regulates nuclear factor kappa-light-chain-enhancer of activated B cells (NF-*κ*B) activation. It has been observed that mutations of the *CARD14* gene and other members of the CARD-containing membrane-associated guanylate kinase protein (CARMA) family may increase the risk of psoriasis and are also linked with CVD. Howes et al. have shown that mutations in the *CARD14* gene increase the risk of psoriasis via the up-regulation of the mucosa-associated lymphoid tissue lymphoma translocation protein 1 (MALT1) paracaspase activity, which is required for optimal NF-*κ*B pathway function and significant expression of the pro-inflammatory genes in the keratinocytes [17]. The authors also suggested that MALT1 inhibitors promote the reduction of skin inflammation triggered by *CARD14* gene mutant variants and therefore may possibly serve as a treatment option in psoriasis. Moreover, *CARD14* gene expression was found not only in keratinocytes but also in lymphatic and aortic endothelial cells, what suggests that *CARD14* mutations may contribute to the psoriasis comorbidities of cardiovascular and systemic origin [18].

### 2.2. Psoriasis and Cardiovascular Risk Factors—Defining Common Pro-Inflammatory Pathways

Many pro-inflammatory cytokines and chemokines are produced locally within psoriatic plaques. Afterwards, their release into the systemic circulation predisposes to CVDs [19]. This risk particularly rises in patients with psoriasis and obesity. Adipose tissue as a source of pro-inflammatory interleukins constitutes a driving force in the development of diabetes, MS, and CVD [20]. The interactions between immunological factors (dendritic cells, cytokines, T-cells, macrophages, chemokines), oxidative stress, adipokines, angiogenesis, endothelial dysfunction, visceral adipose tissue, and up-regulation of pro-inflammatory cytokines as well as concomitant disorders seem to play a crucial role in the exacerbation of psoriasis [21].

### 2.3. ‘Psoriatic March’ Concept

Pro-inflammatory factors overproduction in the course of psoriasis may lead to premature atherosclerosis (called the ‘psoriatic march’ concept) and as a consequence to the other negative effects in the cardiovascular system [22]. The infiltration of sclerotic plaque and psoriatic plaque with common inflammatory molecules was confirmed also in histopathological findings [23]. According to data, patients with psoriasis have 2.67-fold higher odds of developing atherosclerosis in comparison to controls [24]. Moreover, almost 50% of psoriasis patients aged 30–39 years had evidence of subclinical atherosclerosis as compared to 15% of non-psoriasis individuals [24]. These data suggest that psoriasis may affect the inflammatory cascade of atherosclerosis and therefore increase the risk of CVD development. This risk seems to be higher especially among patients with an earlier onset of psoriasis.

## 3. Possible Common Genetic Background of Psoriasis and Concomitant Cardiovascular Risk Factors

The analysis of the current knowledge of the potential genetic links between psoriasis and cardiovascular risk factors involved a thorough literature search of the PubMed database. This article highlights the potential gene candidates that were found to increase cardiovascular risk and were also associated with higher susceptibility to psoriasis. The authors focused on the studies on the genetic pathways evaluated in psoriatic patients. Therefore, some of the well-recognized genetic links of cardiovascular risk factors in the general population were not included in the article.

### 3.1. Genetic Pathways of Primary HT and Psoriasis

Hypertension is a complex disorder. The global prevalence of HT in 2010 was estimated at 31.1% in the general population [25], whereas in patients with mild and severe psoriasis, this index is similar and estimated at 30% [26]. In the course of HT, multiple genetic and environmental factors interact to create the final phenotype. The genetic origin is responsible for the development of 30–50% of HT cases [27]. Some physiological pathways can help reveal gene candidates of the monogenic and less common variants of HT: mineralocorticoid (e.g., *WNK1*, *SLC12A3*, *ATP2B3*), glucocorticoid (e.g., *CYP11B1*, *HSD3B2*, *CYP17A1*), and sympathetic (e.g., *VHL*, *SDHB*, *SDHC*, *SDHD*, *RET*) pathways [28]. Different polymorphisms in angiotensinogen gene (*AGT*) have been reported to influence the rate of *AGT* transcription and therefore AGT serum level. Meta-analysis of the seven case-control studies in the general population showed an association of the angiotensinogen gene polymorphism (*AGT* G-217A) and HT (especially among Asians); however, the authors do not elucidate the polymorphism influence on the psoriasis risk [29]. According to another study, angiotensin-converting enzyme (ACE) insertion/deletion (I/D) gene polymorphism may affect susceptibility to early-onset psoriasis [30], yet its role in HT pathogenesis needs further evaluation due to contradictory study results [31].

Recent studies have shown that the *LNPEP* gene codes the insulin-responsive aminopeptidase, which is identified as an angiotensin IV receptor and is considered as a principal constituent of the renin–angiotensin system. Moreover, the *LNPEP* gene product plays a pleiotropic role in various biological processes, which are closely associated with the pathogenesis of HT, diabetes, and other metabolic consequences i.e., the glucose uptake action mechanism via the insulin-responsive glucose transporter GLUT4 receptor, vasopressin clearance system, and serum sodium levels regulation [32]. GWAS analysis performed by Cheng et al. identified *LNPEP* A763T polymorphism as a potential psoriasis genetic risk factor, and furthermore, the genetic link of concomitance with HT and diabetes. The missense *LNPEP* A763T mutation leads to the peptide function disruption and its down-expression in the skin of psoriatic patients [33].

Endothelial nitric oxide synthase enzyme is the *eNOS* gene product that regulates nitric oxide (NO) production, which is responsible for the regulation of endothelial vasodilation. The alterations of its function are associated with HT and ischemic heart disease pathogenesis. Moreover, NO plays a substantial role in keratinocytes’ growth and differentiation. Three enzyme isoforms (neuronal, nNOS; inducible, iNOS; and endothelial, eNOS) were distinguished [34,35]. A pilot study performed in the Turkish population showed a statistically significant higher T allele prevalence of the *eNOS* Glu298Asp polymorphism in psoriasis patients in comparison to normotensive non-psoriatic healthy volunteers. In addition, *eNOS* Glu298Asp polymorphism was also more frequent in hypertensive psoriatic patients in comparison to normotensive psoriatic patients. These results suggest that *eNOS* Glu298Asp polymorphism may be an independent HT and psoriasis risk factor. However, the authors underscored the need for large-scale studies [36]. On the other hand, Coto-Segura et al. did not demonstrate any association between the *NOS3* Glu298Asp polymorphism, psoriasis risk, and HT in the Spanish population. Instead, the *NOS3* variants −786 T/C and intron 4 VNTR corresponded with higher susceptibility to psoriasis but not with HT and CVD [37]. Moreover, another study revealed the influence of (CCTTT)n pentanucleotide repeat polymorphism in the *iNOS* gene promoter on the greater psoriasis prevalence among the Chinese–Taiwanese population, although it did not evaluate its role in the HT prevalence [38]. Evaluation of the allelic variance within the major psoriasis susceptibility complex HLA (Human Leukocyte Antigen) showed an allele variability in the context of psoriasis phenotype, ethnicity, and possible metabolic disturbances. In the Chinese population, carriers of the HLA-A*02:07 allele showed a positive correlation with nail psoriasis and HT in comparison to healthy controls [39]. This may suggest that certain psoriasis phenotypes could be linked with the concomitant metabolic diseases. Of note, a pilot study on an animal model, concerning IL-17A involved both in the pathogenesis of psoriasis and HT inflammatory model signaling, indicated IL-17 antibodies as a potential treatment target for HT and associated end-organ dysfunction [40].

### 3.2. Genetic Pathways of Insulin Resistance, DM-2, and Psoriasis

Type 2 diabetes is a common disease affecting about 6.28% of the global population, and it is responsible for over 1 million deaths per year [41]. The disease is diagnosed in approximately 10–20% of psoriatic patients [26], showing psoriasis disease-severity dependence on the impaired glucose levels and obesity [42]. Recent robust progress in GWAS of DM-2 revealed >250 associated genomic localizations [43]. The most recognized in the general population susceptibility genes involve *CDKAL1*, *TCF7L2*, *SLC30A8*, and *CDKN2B* (coding for proteins regulating pancreatic islets function, cyclin-dependent kinases, and zinc transporters) with some candidates associated with obesity and insulin-resistance genetic traits (such as *FTO*, *PPAR-γ*, *IRS1*) [44]. The *CDKAL1* gene function has not been resolved yet. Its variant is associated with reduced insulin secretion, leading to DM-2 in the European-ancestry population according to extensive GWAS analysis [45]. Wolf et al. mapped SNPs in the non-coding regions within intron 5 of the *CDKAL1* transcript and revealed its association with higher prevalence of psoriasis, suggesting the putative mutual starting point in the pathogenesis of both disorders [46]. The positive correlation was also confirmed by other researchers [47]. On the contrary, Quaranta et al. underpin the sophisticated *CDKAL1* gene association signals with completely independent gene polymorphisms responsible for DM-2 and psoriasis risk [48]. Furthermore, it is presumed that the *CDKAL1* gene variant may facilitate a positive response to the anti-TNF-α biological treatment among psoriatic patients [49]. TNF-α as a principal pro-inflammatory cytokine is found to be highly overexpressed in the hyperglycemic state and its inhibition (by adalimumab) among non-diabetic psoriatic patients showed an improvement in the insulin sensitivity [50]. However, the preliminary evaluation of the two *TNF-α* promoters’ polymorphisms did not confirm any relevance with higher occurrence of diabetes, HT, and cardiac diseases among psoriatic patients [51].

The GWAS study indicated that *JAZF1* and *ST6GAL1* genes polymorphisms have been both identified as novel causal risk genes for DM-2 in the general population. The *JAZF1* (encoding the protein juxtaposed with another zinc finger protein 1) mutations impair pancreatic B-cells’ development and regeneration [52], whereas the *ST6GAL1* (encoding ST6 beta-galactoside alpha-2,6-sialyltransferase 1 protein) expression influences N-glycosylation pathway changes that lead to diabetes [53]. The study performed by Wang et al. among the Chinese population revealed that *JAZF1* and *ST6GAL1* genes polymorphisms may substantially increase the likelihood of psoriasis and diabetes concomitance, although further investigations upon the exact mutual pathomechanisms are required [54].

Finally, the psoriasis susceptibility loci (*PSORS 2, PSORS3*, and *PSORS4*) have been reported to be closely related to genetic predisposition to DM-2 as well as to MS and CVD [55]. The IL12/23 psoriasis pivotal pathway genes variants (*IL12B* and *IL23R)* also showed some association with DM-2 in a Spanish cohort [56].

### 3.3. Genetic Pathways of Dyslipidemia and Psoriasis

Lipid abnormalities are significantly prevalent among psoriatic patients. Severe psoriasis is associated with approximately 1.4–5.5 times greater risk of dyslipidemia [57]. The pro-atherogenic profile includes decreased high-density lipoprotein (HDL) levels, increased low-density lipoprotein (LDL), very-low-density lipoprotein (VLDL), and triglyceride (TG) levels [58]. Large-scale GWAS research led to the breakthrough discovery of >150 SNPs of European, East Asian, South Asian, and African ancestries, which comprise about 40% of blood lipid levels’ heritability. The pathway analysis showed a complex web of interrelated traits including CVD, DM-2, blood pressure, body mass, and glucose level [59].

The *PCSK9* gene codes for the proprotein convertase subtilisin/kexin type-9 (PCSK9) which plays a chaperone protein role in clearance of the LDL receptors, resulting in the serum LDL concentration amplification—a starting point of the cardiovascular events. The PCSK9 is also involved in the chronic pro-inflammatory pathway promotion. There is evidence that the *PCSK9* mRNA overexpression in the psoriatic lesions leads to the keratinocyte hyperproliferation, and thus, disease exacerbation [60]. There is also evidence for the eminent role of the PCSK9 in the psoriasis and cardiometabolic syndrome linkage [61]. Given these findings, the latest generation of the PCSK9 inhibitor may lead to a novel approach to the psoriasis treatment.

GWAS studies of the dyslipidemia trait among the Caucasian population did not show any loci overlap within the major psoriasis susceptibility complex (*MHC*) region, therefore indicating a separate genetic origin from psoriasis [62]. Shen et al. found an association in the Asian study group of the HLA C*01:02 allele with the potential risk of dyslipidemia among psoriatic patients, although the authors highlighted the need for the study replication in a larger cohort [39].

Recent analysis of the transcriptome profiling has led to the discovery of the pathophysiological connection between psoriasis and dyslipidemia via the IL-17A cytokine pathway. The pivotal psoriasis IL-17A signaling up-regulates the cellular cholesterol concentrations, leading to its accumulation in keratinocytes. Consecutively, the exerted reciprocal signal suppresses the cholesterol and fatty acid production by impeding the sterol regulatory element binding proteins (SREBP) group of transcriptional factors that control the lipid homeostasis [63].

### 3.4. Genetic Pathways of Obesity, Abnormal Body Mass Index (BMI), and Psoriasis

Obesity is a key psoriasis comorbidity leading to a higher prevalence of CVDs. Epidemiological studies reported a variable frequency of obesity in psoriasis patients ranging from 15 to 30%; however, most studies recruited individuals of Caucasian ancestry [26]. Body weight and obesity are highly dependent on genetic heritability, approximately in 65–80% [64]. Obesity is an extremely multifactorial disease; therefore, even in the GWAS era, the majority of its genetic origin remains undetermined. More than 870 SNPs collectively determine about 3–5% of the “common”/multifactorial obesity variability [65]. Monogenic obesity results from highly infrequent single genes mutations (such as *LEP*, *POMC*); however, some of them also demonstrated an association with polygenic obesity (e.g., *MC4R*, *FTO*) [65]. They encompass a complex system of body mass regulation: appetite and satiety neurological trail, insulin secretion, adipogenesis, energy and lipid metabolism, gene–environment interactions (dietary and physical activity). Furthermore, recent studies revealed that obesity-related genetic loci overlap with various obesity-related diseases pathways (HT, diabetes, cardiovascular disease) [66]. The *FTO* gene product is a protein (2-oxoglutarate-dependent nucleic acid demethylase) responsible for DNA repair and energy homeostasis control, which is mainly expressed in hypothalamus nuclei and therefore exerts an influence on fat mass and obesity. Coto-Segura et al. demonstrated that *FTO* gene polymorphism was associated with higher mean BMI value and augmented the risk of obesity among psoriatic patients in the Spanish population; however, the prevalence of the risk allele was comparable to the general population of that region [67]. These findings were confirmed by Tupikowska-Marzec et al., who observed that the *FTO* gene variant was associated with obesity, and moreover, greater psoriasis severity and obesity-related insulin resistance among Polish population [68]. Common polymorphic variants near the *MC4R* gene, which encodes the hypothalamic melanocortin 4 receptor, are widely recognized genetic risk factors linked with early-onset severe obesity [69]. To our knowledge, only one study evaluated the effect of *MC4R* gene polymorphism on the body composition in the context of psoriatic patients of Romanian ethnicity. The researchers imply that the mutations near the *MC4R* gene region significantly increased the risk of obesity, diabetes mellitus, and psoriatic arthritis in the presented study group, although large-scale prospective studies are required [70].

Adipose tissue is an endocrine organ that secrets various bioactive molecules, mainly adipokines. The excessive amount of adipose tissue is associated with decreased levels of anti-inflammatory adiponectin (*ADIPOQ* gene) and increased levels of pro-inflammatory leptin and leptin receptors (*LEP*, *LEPR* genes) in psoriasis [71]. The investigation of the genetic link between adipokines genes and obesity among psoriatic patients brought contradictory results [72]. However, Mitsuyama et al. found a positive correlation between the leptin mRNA expression in subcutaneous adipose tissue and the obesity level among psoriatic patients, and furthermore, aggravated psoriasis severity and elevated serum leptin levels [73]. The study results clarify the influence of obesity on the psoriasis clinical course. Nonetheless, they do not elucidate the role of leptin genetic background on the concordance of psoriasis and obesity. An investigation carried out on the imiquimod-stimulated psoriasis mouse model with genetically induced leptin deficiency revealed that leptin-deficient mice presented alleviated disease phenotype and down-regulated the IL-17A and IL-22 mRNA expression in the skin, and moreover, clinical signs of malnutrition [74].

Peroxisome proliferator-activated receptor gamma gene (*PPAR-γ*) is a well-known genetic regulator of the adipocyte differentiation and intracellular insulin-signaling pathway. The polymorphic variations of its function lead to metabolic disturbances such as obesity [75]. In psoriasiform mice skin, the expression of PPAR-*γ* is reported to be down-regulated, which is hypothesized to activate chronic pro-inflammatory cellular pathways and immunological response that is associated with psoriasis pathogenesis and comorbidities prevalence. However, the *PPAR-γ* gene was not identified as a psoriasis susceptibility gene locus [76]. Still, there is scarce data concerning the *PPAR-γ* gene influence on the higher obesity incidence among psoriatic patients. A case-controlled study among 45 Egyptian psoriatic patients showed the statistically significant association of the Ala allele of the *PPAR-γ* gene (Pro12Ala polymorphism) with obesity and psoriasis occurrence, although no correlation with MS incidence was found [77]. Additionally, a pilot study carried out in the obese psoriatic patients demonstrated the anti-inflammatory skin effect of the orally administered PPAR-*γ* agonist (pioglitazone), which was confirmed by the immunohistochemical detection [78].

In a case-control study on the Chinese population, the major psoriasis susceptibility locus HLA-Cw*06 was significantly correlated with BMI, waist-to-hip-ratio (WHR), and obesity. Obese individuals with the presence of the HLA-Cw*06 allele had a 35-fold greater risk of psoriasis in comparison to allele negative and non-obese study participants [79]. No studies were conducted on the Caucasian population.

Further insight into the elaborated psoriasis cytokine axis showed an impact of patients’ excessive body mass (defined both as visceral and overall adiposity) on the higher incidence risk of psoriasis among carriers of the *IL12B* gene polymorphism. This confirms the impact of the gene–environment interaction on the elevated susceptibility to psoriasis [80].

### 3.5. Genetic Pathways of MS and Psoriasis

The large-scale meta-analysis of the population-based studies carried out in the study group of 40,000 psoriatic patients demonstrated the strong link between psoriasis and MS, with more than two-fold susceptibility risk in comparison to the general population. Moreover, the prevalence of the comorbidity was psoriasis severity-dependent [81]. The MS genetic susceptibility was confirmed in a familial and twin study of the Caucasian ancestry [82]. To date, with the help of the GWAS technology, the clusters of the MS genetic traits were identified with the heritability range of 13–27% [83]. Due to the fact that MS comprises of autonomous components (e.g., fasting glucose abnormalities, HT, dyslipidemia, and obesity) it is believed that the co-existence of several genetic risk clusters rather than a separate genetic locus is responsible for its genetic etiology [84]. However, the GWAS research of the Finnish population revealed the strong association of the *APOA1/C3/A4/A5* gene cluster with MS, which indicates that the alterations in the lipid metabolism pathways are the crucial components of the MS genetic etiology [85]. The post-GWAS analysis of the shared genetic architecture between psoriasis, MS, and coronary artery disease (CAD) did not show any association of the *MHC* locus with the locus of MS and CAD [86]. So far, there are no studies providing evidence about the shared genetic locus of MS and psoriasis; thus, the association between these two is not fully determined. On the other hand, Abdel Hay et al. showed that the leptin gene polymorphism (*LEP* G-2548A), coding an adipokine upregulated both in obesity and psoriasis, was associated with genetic predisposition to psoriasis and MS among Egyptian patients and could serve as a marker of the cardiovascular comorbidities [87]. In contrast, the case-control study on the Turkish population did not confirm the association between the *LEP* G-2548A polymorphism and the incidence of psoriasis [88].

### 3.6. Genetic Pathways of Depression and Psoriasis

Depression is considered as an important risk factor of CVD and may contribute to poor cardiovascular outcomes [89]. Mood disorders and major deterioration of quality of life are reported to be common among psoriatic patients, with the prevalence of up to 60% [57]. Twin studies proved that the heritability of depression ranged from 31 to 42% [90]. A huge power GWAS study on the genetic origin of depression proved a highly polygenic trait with over >100 independent SNPs identified related mainly to the neurotransmission and stimuli response pathways within the cortical brain regions. Moreover, the analysis of the shared genetic pathways showed an overlap with traits associated with dyslipidemia, obesity, and CVD, which are common psoriasis comorbidities [91]. According to Aberra et al., self-reported depression among psoriatic patients is linked with increased vascular inflammation as well as coronary plaque burden and therefore may promote subclinical atherosclerosis [92]. The pro-inflammatory cytokine circulation theory overlaps with depression, suicidality, and psoriasis. Mood disorders may originate either from the psoriasis burden or the direct pro-inflammatory background with the important role of shared cytokine e.g., TNF-α, IL-17, IL-1, and IL-6 [93,94]. This theory is supported by the identification of the immune-derived genetic background and the mRNA expression that influences depression risk, clinical phenotype, and drug therapy response [95]. Murphy et al. identified the differentially methylated region nearby the *PSORS1C3* gene on chromosome 6 associated with the major depressive disorder and suicide in brain samples of Canadian ancestry [96]. The gene product is putatively related to the immune system regulation; however, further insights are required. The *PSORS1C3* locus plays a fundamental role in psoriasis susceptibility and therefore might represent the potential genetic link between psoriasis and depression [96]. The methylation mutation of the *PSORS1C3* locus was confirmed by Lapsley et al. in the investigation of the depressive disorder among Caucasian young adults [97]. Moreover, further evidence of the genetic overlap was explored due to methylation gain at the epidermal differentiation complex of the late cornified envelope (*LCE* gene cluster), which has a considerable impact on psoriasis development. The pyrosequencing study revealed the role of *MIR4520A/B* polymorphism in depression, which is also linked with psoriasis pathophysiology [97].

The possible genetic links between psoriasis and cardiovascular risk factors are presented in Table 2.

### 3.7. Genetic Linkage between CVD and Psoriasis

Cardiovascular disease is one of the most frequently observed psoriasis comorbidities, although the epidemiological studies describe a wide discrepancy in its prevalence—from 2 to 18% of psoriasis patients [26]. The prevalence of CVD among psoriatic patients may result from the co-occurrence of the cardiovascular risk factors such as HT, obesity, diabetes, and dyslipidemia or could share the pathways as the differentially expressed genes (DEGs) [103]. To date, the studies identified over >50 GWAS loci of the CAD and revealed strong additive associations with the *APOE*, *PCSK9*, and *NOS3* [104]. The investigation among the European population showed the association between the genotypes of the ε2/ε3/ε4 alleles of the *APOE* gene, encoding apolipoprotein E, and the higher LDL cholesterol levels, which correspond with higher CAD risk and stroke [105]. The Saudi Arabia study evaluated the linkage between the *APOE* gene variants and psoriasis and found that frequencies of the ε2 and ε4 alleles and genotypes ε3/ε4 and ε3/ε2 were considerably higher in psoriasis patients in comparison to healthy controls. Moreover, the results corresponded with elevated serum lipid levels [100]. Finally, Lu et al. conducted a meta-analysis of four GWAS cohorts and revealed a possible association in genes of the psoriasis and CAD: *FUT2* encoding an alpha-(1,2) fucosyltransferase, *UBE2L3* encoding an ubiquitin-conjugating enzyme involved in cell proliferation and immune function, and *SH2B3* encoding the adaptor protein that has a pleiotropic signaling role in regulating lymphocyte differentiation [102]. The authors suggested that psoriasis patients are enriched for some genetic variants in these regions and therefore may be predisposed to a higher risk of HT, dyslipidemia, and CVD.

## 4. Summary

There is mounting evidence that the co-occurrence of psoriasis and disorders increasing the risk of CVD might originate from the pleiotropic mechanisms of interactions with many genetic pathways. The gaps in the understanding of the clinical prevalence of psoriasis and its comorbidities may be caused by the multiple pleiotropic disease loci localized in intronic regions, which are highly heterogenous disease traits composed of multiple loci of small effects or poorly captured disease etiopathology and biological processes. Furthermore, the genetic bondage might also not result from shared genetic background but rather from the gene–environment interactions or epigenetic alterations. Still, further studies into the shared genetic background are needed to elucidate those associations.

Possible clinical implications and benefits from the future genetic studies include early patients’ screening and education leading to prophylaxis and treatment, primary disease control and better life quality, assessment of the response to treatment (pharmacogenomic studies, precision medicine—long distance future), and decrease in cardiovascular risk as well as decrease in mortality rate.

## Figures and Tables

**Table 1:** Comorbid diseases in psoriasis.

Comorbidities in Psoriasis	
**Physical**	psoriatic arthritis hypertension diabetes dyslipidemia obesity metabolic syndrome cardiovascular disease inflammatory bowel disease nonalcoholic fatty liver disease chronic obstructive pulmonary disease uveitis Parkinson’s disease lymphomas osteoporosis celiac disease erectile dysfunction sleep apnea
**Psychological**	depression suicidality anxiety smoking alcoholism
**Treatment related**	hypertension dyslipidemia hepatotoxicity nephrotoxicity skin neoplasms

**Table 2:** Genetic linkage of psoriasis and cardiovascular risk factors.

Chromosome	SNP	Nearby Gene	Gene Product	Minor/Major Allele	Findings in Psoriasis	SAMPLE SIZE	Population	Authors	Cardiovascular Associations
17	rs4341	*ACE*	angiotensin I converting enzyme	287-base pair insertion ⁄deletion (I ⁄D)	homozygosity for the ACE I allele was considerably more prevalent in patients with early-onset psoriasis in comparison to the control subjects	207 patients and 182 controls	Central European	Weger et al. (2007) [30]	might interact with angiotensinogen gene haplotypes which are linked with HT (Tsai et al. (2003)) [31]
5	rs2303138	*LNPEP*	leucyl and cystinyl aminopeptidase	A/T	significantly down-regulated in the involved skin of psoriasis patients compared with the skin of the controls	8339 patients and 12,725 controls	Asian	Cheng et al. (2014) [33]	important component of the renin–angiotensin system responsible for primary HT pathogenesis via vasopressin clearance and serum sodium regulation (Nakada et al. (2011)) [32] affects glucose uptake via the interaction of the insulin receptor signaling with the insulin-responsive glucose transporter GLUT4 (Shibata et al. (2007)) [98]
7	rs1799983	*eNOS Glu298 Asp*	endothelial nitric oxide synthase	T/G	T allele greater frequency was found to be associated with higher incidence of psoriasis itself, and moreover, HT among psoriatic patients	75 patients and 55 controls	South European	Ogretmen et al. (2014) [36]	triggering factor of the endothelial disfunction, vasodilation disruption and furthermore thrombocyte aggregation via blood flood impeding (Moncada et al. (1993)) [34]
6		*HLA-A*	major histocompatibility complex, class I, A	HLA-A 07*02	carriers of the allele had a greater susceptibility to HT and psoriasis (in particular nail psoriasis)	120 patients and 130 controls	Asian	Shen et al. (2019) [39]	No data
6	rs6908425	*CDKAL1*	CDK5 regulatory subunit-associated protein 1-like 1		associated with greater incidence of psoriasis	1256 patients and 2938 controls	North European	Wolf et al. (2008) [46]	gene function remains unresolved; however, the polymorphism is associated with the pathogenesis of DM-2 via the impaired insulin response and CVD (Steinthorsdottir et al. (2007)) [45]
7	rs849135	*JAZF1*	JAZF zinc finger 1	A/G	newly identified susceptibility gene for psoriasis	4483 psoriasis and 6030 controls	Asian	Wang et al. (2017) [54]	pancreatic beta-cell function and glucose metabolism impairment which leads to DM-2 (Grarup et al. (2008)) [99]
3	rs16861329	*ST6GAL1*	ST6 beta-galactoside alpha-2,6-sialyltransferase 1	T/C	newly identified susceptibility gene for psoriasis	4483 psoriasis and 6030 controls	Asian	Wang et al. (2017) [54]	involved in the process of N-glycosylation of proteins that alters their structure and function and are described to take part in type 1 and 2 diabetes (Rudman et al. (2019)) [53]
6		*HLA-C*	major histocompatibility complex, class I, C	HLA-C 01*02	greater occurrence among psoriasis patients, elevated risk of dyslipidemia in psoriasis	120 patients and 130 controls	Asian	Shen et al. (2019) [39]	No data
16	rs9930506	*FTO*	alpha-keto-glutarate dependent dioxygenase	G/A	homozygotes for the G allele psoriatic patients had a higher mean BMI index	413 patients and 210 controls	South European	Coto-Segura et al. (2014) [67]	major influence of fat mass increase and obesity (Su et al. (2017)) [66]
16	rs9939609	*FTO*	alpha-ketoglutarate dependent dioxygenase	A/T	among psoriatic patients the polymorphism presence carried greater risk of obesity (increased BMI) and diabetes (increased insulin concentrations)	197 psoriatic patients	Central European	Tupikowska-Marzec et al. (2019) [68]	major influence of fat mass increase and obesity (Su LN, et al. (2017)) [66]
18	rs17782313	*MC4R*	hypothalamic melanocortin 4 receptor		greater expression associated with obese psoriasis patients in comparison to non-obese psoriasis patients	82 psoriasis patients	Central European	Voiculescu et al. (2018) [70]	widely recognized genetic risk factor for early-onset severe obesity (Loos et al. (2009)) [69]
8		*PPAR-γ*	peroxisome proliferator-activated receptor-γ	Pro12Ala	significant link between the risk of psoriasis itself and additionally obesity among those patients	45 psoriasis patients and 45 controls	Middle East	Seleit et al. (2019) [77]	adipocyte differentiation and intracellular insulin-signaling pathway, which promotes obesity (Prakash et al. (2012)) [75]
6		*HLA-C*	major histocompatibility complex	HLA-C 06*02	major psoriasis genetic risk factor allele, correlation with a significant body mass increase and elevated WHR ratio	466 patients and 177 controls	Asian	Jin et al. (2008) [79]	No data
7	rs7799039	*LEP*	leptin (adipokine)	G2548A	polymorphism carriers had a greater prevalence of psoriasis, MS, and higher plasma leptin	94 patients and 100 controls	Middle East	Abdel Hay et al. (2011) [87]	higher plasma leptin is associated with greater obesity risk and also psoriasis (Gerdes et al. (2011)) [71]
19		*APOE*	apolipopro-tein E	ε2 and ε4 alleles	greater allele frequencies correlated with higher risk of psoriasis and higher serum cholesterol, triglycerides, and LDL levels among psoriatic patients	94 patients and 200 controls	Middle East	Al Harthi et al. (2014) [100]	apolipoprotein E influences lipid metabolism processes, which promote atherosclerosis, and thus, lead to CVD (Bennet et al. (2007)) [101]
19	rs492603	*FUT2*	alpha-(1,2)fucosyltran-sferase	A/G	higher risk of dyslipidemia among psoriatic patients (mainly increase triglyceride levels)	4482 patients and 7463 controls	Multi	Lu et al. (2013) [102]	No data
22	rs181362	*UBE2L3*	ubiquitin-conjugating enzyme	T/C	higher risk of dyslipidemia among psoriatic patients (mainly decreased HDL-C)	4482 patients and 7463 controls	Multi	Lu et al. (2013) [102]	No data
12	rs3184504	*SH2B3*	SH2B adaptor protein 3	T/C	higher risk of CAD and elevated systolic and diastolic blood pressure measurements	4482 patients and 7463 controls	Multi	Lu et al. (2013) [102]	No data

## Data Availability

Not applicable.

## Abbreviations

ACE	angiotensin-converting enzyme
AGT	angiotensinogen
BMI	body mass index
CAD	coronary artery disease
CARD14	caspase recruitment domain-containing protein 14
CARMA	CARD-containing membrane-associated guanylate kinase protein
CVD	cardiovascular disease
DEG	differentially expressed genes
DM-2	type 2 diabetes mellitus
DNA	deoxyribonucleic acid
eNOS	endothelial nitric oxide synthase
GLUT4	insulin- responsive glucose transporter receptor
GWAS	genome wide association study
HDL	high-density lipoprotein
HLA	human leukocyte antigen
HT	hypertension
IL	interleukin
iNOS	inducible nitric oxide synthase
LDL	low-density lipoprotein
lncRNA	long non-coding ribonucleic acid
MALT1 MHC	mucosa-associated lymphoid tissue lymphoma translocation protein 1 major histocompatibility complex
mRNA	messenger ribonucleic acid
MS	metabolic syndrome
NF-*κ*B nNOS	nuclear factor kappa-light-chain-enhancer of activated B cells neuronal nitric oxide synthase
NO	nitric oxide
PCSK9	proprotein convertase subtilisin/kexin type-9
PPAR-*γ*	peroxisome proliferator-activated receptor gamma
SNP	single nucleotide polymorphism
SREBP	sterol regulatory element binding protein
TG	triglyceride
Th	T-helper lymphocyte
TNF-α	tumor necrosis factor alpha
VLDL	very-low-density lipoprotein
WHR	waist-to-hip-ratio
